# Autophagy and Exosomes in the Aged Retinal Pigment Epithelium: Possible Relevance to Drusen Formation and Age-Related Macular Degeneration

**DOI:** 10.1371/journal.pone.0004160

**Published:** 2009-01-08

**Authors:** Ai Ling Wang, Thomas J. Lukas, Ming Yuan, Nga Du, Mark O. Tso, Arthur H. Neufeld

**Affiliations:** Forsythe Laboratory for the Investigation of the Aging Retina, Department of Ophthalmology, Northwestern University School of Medicine, Chicago, Illinois, United States of America; University of Oldenburg, Germany

## Abstract

Age-related macular degeneration (AMD) is a major cause of loss of central vision in the elderly. The formation of drusen, an extracellular, amorphous deposit of material on Bruch's membrane in the macula of the retina, occurs early in the course of the disease. Although some of the molecular components of drusen are known, there is no understanding of the cell biology that leads to the formation of drusen. We have previously demonstrated increased mitochondrial DNA (mtDNA) damage and decreased DNA repair enzyme capabilities in the rodent RPE/choroid with age. In this study, we found that drusen in AMD donor eyes contain markers for autophagy and exosomes. Furthermore, these markers are also found in the region of Bruch's membrane in old mice. By *in vitro* modeling increased mtDNA damage induced by rotenone, an inhibitor of mitochondrial complex I, in the RPE, we found that the phagocytic activity was not altered but that there were: 1) increased autophagic markers, 2) decreased lysosomal activity, 3) increased exocytotic activity and 4) release of chemoattractants. Exosomes released by the stressed RPE are coated with complement and can bind complement factor H, mutations of which are associated with AMD. We speculate that increased autophagy and the release of intracellular proteins via exosomes by the aged RPE may contribute to the formation of drusen. Molecular and cellular changes in the old RPE may underlie susceptibility to genetic mutations that are found in AMD patients and may be associated with the pathogenesis of AMD in the elderly.

## Introduction

Age-related macular degeneration (AMD) is a progressive degeneration of the macula of the retina, usually bilateral, leading to a severe decrease in fine vision and a central scotoma in the elderly [1]. AMD is broadly classified as either dry (non-neovascular) or wet (neovascular) [2]. The dry form of AMD, which is characterized by drusen in the macula, is more common and accounts for about 85 to 90% of patients with AMD. Patients with dry AMD have a substantial risk of developing wet AMD.

Drusen, which may occur throughout the retina, contain extracellular deposits of biological material adhering to Bruch's membrane between the retinal pigment epithelium (RPE) and the choriocapillaris. The proteins found in drusen are an admixture of blood proteins, extracellular proteins and intracellular proteins [3]. Although there has been much attention to the blood proteins (eg complement and CFH) in drusen, there have been few studies on the intracellular proteins and how they may participate in the formation of drusen. Notably, the intracellular proteins in drusen are at least partially intact in that they can be identified by immunohistochemistry and proteomics. We have investigated the conditions and pathways by which intracellular proteins in the RPE become extracellular and, perhaps, end up in drusen.

Our laboratory has previously demonstrated increased mtDNA damage and decreased DNA repair enzyme capabilities in the RPE/choroid with age [4]. Defective mitochondria are associated with aging and may underlie or increase the susceptibility to a variety of neurodegenerative diseases [5]. Proteomic studies indicate changes in mitochondria of the RPE at progressive stages of AMD [6]. Defective mitochondria and other organelles are cleared from the cell by a process known as autophagy in which fusion of damaged material with lysosomes and digestion is the disposal pathway [7]. These processes should keep post-mitotic cells healthy and functional [8]. Nevertheless, increased mtDNA damage in the aging RPE may be associated with increased autophagy.

The RPE, which is adjacent to the photoreceptors and rests on Bruch's membrane, phagocytoses and digests the distal parts of the outer segments of the photoreceptors each day [9], [10]. As a consequence of the daily task of digesting photoreceptor outer segments (POS) throughout life, the lysosomal apparatus of the post-mitotic RPE cells must process and eliminate significant loads of material. In the RPE of old eyes, the physiological lysosomal load may be further increased by autophagy to remove damaged mitochondrial material. With age, there may be insufficient digestion of damaged macromolecules and organelles by the RPE, which will lead to progressive accumulation of biological “garbage”, such as lipofuscin, and release of partially digested or undigested materials. Thus, we hypothesize that the increased mtDNA damage associated with normal aging leads to altered functional capacities of the RPE. Furthermore, age-related changes in cellular functions of the RPE may lead to release of intracellular proteins that contribute to the formation of drusen. These changes with age may provide the background for a genetic mutation to cause the pathophysiological and immunological events leading to the development of drusen in dry AMD. Here, we provide the first evidence for increased autophagy and increased exocytotic activity in the aged RPE and the presence of autophagy and exosome markers in drusen of AMD patients.

## Methods

### Donors

Two independent groups of AMD cases and age-matched controls were used for this study. Twelve eyes from six donors with no history of AMD were obtained from the National Disease Research Interchange. Four eyes from two donors with histories of AMD were obtained from the National Disease Research Interchange. Every pair of eye used had the signed consent of the patient, according to the tenets of the Declaration of Helsinki. In addition, eight eyes from eight donors with well-documented AMD were retrieved from the Mark O.M. Tso Ophthalmic Pathology Laboratory at the Wilmer Eye Institute, Johns Hopkins University School of Medicine. The use of these human tissues in our laboratory is exempt from IRB approval.

### Mouse eye tissues

Frozen eyes of male C57BL/6 mice from young (4 mos) and old (24∼28 mos) animals were obtained from the tissue bank of the National Institute on Aging. All experimental protocols were in compliance with the National Institutes of Health guidelines and were approved by the Center for Comparative Medicine Committee at Northwestern University.

### Immunohistochemistry

The eyes were fixed in buffered formaldehyde, 4%, were washed in phosphate-buffered saline (PBS, pH 7.4), embedded in paraffin and sectioned. The tissue sections were deparaffined with xylene and a series of graded ethanol steps and rehydrated in PBS. The tissues were heated at 95°C to 99°C in citrate buffer (pH 6.0) for 20 min for antigen retrieval. The sections were blocked with normal goat serum, 5%, for 20 min and incubated at 4°C overnight with a first primary antibody, a rabbit anti-Atg5 (1∶50, Novus Biologicals), mouse anti-CD63 (1∶50, Abcam), goat anti-C3 (1∶50, Santa Cruz). Primary antibody was omitted in the negative control. After several washes, tissue sections were incubated with the secondary antibody, Alexa Fluor 568 goat anti-rabbit IgG (1∶1000, Molecular Probes) for 1 hr at room temperature. After washing with PBS, the slides were mounted with Vectorshield containing DAPI (Vector Laboratory) and observed using confocal microscopy.

### Western blot

Tissues or ARPE-19 cells were lysed in buffer (20 mM HEPES, pH 7.0, 10 mM KCl, 2 mM MgCl_2_, 0.5% Nonidet P-40, 1 mM Na_3_VO_4_, 1 mM PMSF, and 0.15 U ml^−1^ aprotinin) and homogenized. Protein concentrations were determined using the Bradford colorimetric assay. Thirty micrograms of each protein lysate were loaded in each lane in sample buffer (2% SDS, 10% glycerol, 0.001% bromophenol blue, 1% DTT, and 0.05 M Tris-HCl, pH 6.8), separated on 10% SDS–PAGE (Invitrogen), and transferred to a PVDF membrane (Millipore, Temecula, CA). The blots were blocked with 5% nonfat milk in PBS and incubated with mouse anti-Atg12 (1∶500, GeneTex), rabbit anti-LC3B (1∶1000, Abcam), rabbit anti-Cathepsin D (1∶4000, GeneTex), goat anti-CD63 (1∶500, Santa Cruz), mouse anti-LAMP2 (1∶500, Santa Cruz), followed by peroxidase-conjugated donkey anti-goat IgG (1∶15,000; Santa Cruz), goat anti-mouse IgG (1∶10,000) or donkey anti-rabbit IgG (1∶15,000) for 1 hr at room temperature. Finally, the blots were developed by enhanced chemiluminescence (ECL) (Pierce) on Hyperfilm (Amersham). The immunoblots were scanned and relative band density was determined using ImageJ (National Institutes of Health, Bethesda, MD). The densities were normalized to β-actin and analyzed by a standard two-tailed t-test using GraphPad Prism.

### APRE-19 cell culture

ARPE-19 cells are a transformed human RPE cell line. ARPE-19 cells were purchased from American Type Culture Collection (ATCC, Manassas, VA) and maintained in DMEM/F12 with 10% fetal bovine serum (FBS), according to published methods [11]. ARPE-19 cells were used as indicated below.

### In vitro autophagy model: Rotenone induced damage of mtDNA

To study the autophagy process that occurs in response to damage of mtDNA, we established an in vitro autophagy model system using rotenone, a widely used inhibitor of mitochondrial complex I. Rotenone (Sigma) was used to damage mtDNA. Reactive oxygen species (ROS) generation by rotenone is dependent upon the presence of mitochondria [12], [13] and complex I [14]. When rotenone interacts with complex I in mitochondria, ROS are formed [12], [13]. We determined the low concentrations of rotenone that damaged mtDNA in ARPE-19 cells, without loss of ATP or CoEnzyme Q (CoQ) levels and with no effect on cell viability, as described below.

### Viability assay

The cell viability assay was done based on a published method [15]. Briefly, ARPE-19 cells were seeded at a density of 8000 cells/well onto 96-well plates. One day after seeding, the plate were treated with media containing 0, 0.08, 0.16, 0.31, 0.63, 1.25, 2.5, 5, 10, 20, 40 µM rotenone. After the cells were treated for 24 hr, the cell viability was quantified by MTT assay (Promega, Madison, WI), following the manufacturer's instructions. Briefly, the wells were washed with normal culture media and incubated with MTT for an additional 4 hr at 37°C. Absorbance at 570 nm was determined using a Microplate Reader (Model 680; Bio-Rad, CA). All assay points were determined in triplicate, and all experiments were repeated three times.

### Quantification of ATP levels in vitro

The ATP levels in the ARPE-19 cells exposed to 0, 0.08, 0.16, 0.31, 0.63, 1.25, 2.5, 5, 10, 20, 40 µM rotenone were measured with a luciferin-luciferase assay kit (ATPlite, Perkin Elmer Life Science). A calibration curve for ATP concentrations was obtained for each experiment by using the same batch of luciferin-luciferase reagents. Briefly, RPE cells in a 96 well format were treated with a cell lysing solution which raises the pH of the cell culture media and inactivates endogenous ATPases. The subsequent addition of the substrate solution (luciferase-luciferin) lowers the pH to a suitable level so that the reaction can occur. Plates were sealed and incubated at room temperature for 10 min and the luminescence was measured, according to the manufacturer's instructions.

### Quantification of CoEnzyme Q levels in vitro

The general method for measuring endogenous CoQ levels is based upon detection by mass spectrometry [16], [17]. We adapted this method for use on ARPE-19 cell pellets. After exposure to rotenone as above, cell pellets (∼5×10^−6^ cells) were re-suspended in 0.1 ml of water and homogenized (2×5 sec) with a small motorized pestle (Kontes) in a 1.7 ml tube. Then, 450 µl of isopropanol was added and the mixture homogenized again and then centrifuged (14000 rpm, 5 min) in a table-top microfuge. The supernatant was removed to a glass tube to which 20 ng of a synthetic α tocopherol analog was added as an internal standard. The resulting pellet was re-extracted with 450 ul of isopropanol and centrifuged again. Combined supernatants were evaporated to dryness using a Speed-Vac concentrator. The residue was re-suspended in 50 µl of 55:45:0.05% isopropanol/methanol/formic acid (v/v/v) and transferred to a small tube. The mixture was centrifuged to remove insoluble material and the supernatant analyzed immediately by liquid chromatography-mass spectrometry (LC-MS) for CoQ content.

CoQ was measured using an Agilent 1100 XCT LC-MS. This analysis used a reversed phase HPLC column (YMC 2.1×100 mm C18) thermostated at 40°C. The running eluant was 55:45:0.05 isopropanol/methanol/formic acid containing 1 mM methylamine at 150 µl/min. Methylamine was used to increase the sensitivity of the mass spectrometer for CoQ by producing single methylammonium ion adducts instead of a mixture of protonated (MH^+^) or sodium (MNa^+^) ion species [16]. The electrospray source of the ion trap mass spectrometer was set in ESI mode with a 20 psi nitrogen nebulizer flow and capillary temperature at 280°C. The inlet capillary voltage was −2800 V. Helium was used as the collision gas. The instrument was programmed to scan from 100 to 1200 m/z at 3400 m/z/sec. The m/z range 861–920 was targeted for MS/MS operations to fragment the target ions of CoQ. Of the two major forms of CoQ (CoQ_9_, CoQ_10_ subscript refers to the number of isoprene units) found in mammalian tissues, only the CoQ_10_ form was detected in RPE cells. Also as expected, the major peak in the MS/MS spectra of CoQ_10_ was the MH^+^ ion (m/z 863) due to loss of the methylammonium. CoQ standards spanning a range of 100 to 2000 pg were used to calibrate the instrument peak height response for the m/z 894 ion (M-CH_3_NH_2_^+^) of CoQ.

### Long Extension-Polymerase Chain Reaction (LX-PCR)

LX-PCR [18] was performed on rotenone treated ARPE-19 cells (described above). Genomic DNA was isolated with DNeasy Blood & Tissue Kit (Qiagen). The quantitation of the purified genomic DNA, as well as of PCR products, was performed fluorometrically using the PicoGreen dsDNA reagent (Invitrogen). LX-PCR was performed with the GeneAmp XL PCR system (Applied Biosystems), which uses rTth DNA Polymerase XL enzyme designed to amplify target DNA sequences up to about 40 kB. The amounts of primers were 20 pmol and the Mg^2+^ concentration was 1.3 mM. The six pairs of PCR primers employed in this study are given in Table 1.

**Table 1:** DNA primers used for LX-PCR

**16.2-kb mitochondria fragment**
15149 5′-TGA GGC CAA ATA TCA TTC TGA GGG GC-3′ Sense
14841 5′-TTT CAT CAT GCG GAG ATG TTG GAT GG-3′ Antisense
**7.5 Kb mitochondrial fragment**
15149 5′-TCT AAG CCT CCT TAT TCG AGC CGA -3′ Sense
5999 5′-TCT AAG CCT CCT TAT TCG AGC CGA-3′ Antisense
**Short fragment of mtDNA (221 bp)**
14620 5′-CCC CAC AAA CCC CAT TAC TAA ACC CA-3′ Sense
14841 5′-TTT CAT CAT GCG GAG ATG TTG GAT GG-3′ Antisense
**13.5-kb fragment from the 5**′ **flaking region near the β-globin gene**
48510 5′-CGA GTA AGA GAC CAT TGT GGC AG-3′ Sense
62007 5′-GCA CTG GCT TAG GAG TTG GAC T-3′ Antisense
**12.2 Kb region of the DNA polymerase gene β**
2372 5′- CAT GTC ACC ACT GGA CTC TGA AC -3′ Sense
*3927 5* ′*- CCT GGA GTA GGA ACA AAA TTG* * CT -3*′ *Anti-sense*

All the protocols were initiated by a hot start (75°C, 2 min) prior to addition of rTth enzyme. For amplification of the long fragment of mtDNA, the standard thermocycler program included initial denaturation at 94°C for 1 min, 26 cycles for 15149/14841 or 19 cycles for 15149/5999 of 94°C 15 sec, 65°C 12 min, with final extension at 72°C for 10 min. To amplify a short mtDNA fragment (221 bp) the same program as 15149/14841 was used except that extension temperature was 60°C. To amplify a long nDNA fragment, the thermocycler profile included initial denaturation at 94°C for 1 min, 27 cycles for β-globin or 26 cycles for β-polymerase of 94°C 15 sec, 65°C 12 min, with final extension at 72°C for 10 min. To amplify a short nDNA fragment (84 bp), the same program as β-globin was used except that extension temperature was 60°C.

DNA damage was quantified by comparing the relative efficiency of amplification of large fragments of DNA (16.2 and 7.5 KB from mtDNA and 13.5 and 12.2 KB for nDNA) and normalizing this to the amplification of smaller (221 bp and 84 bp) fragments. The template DNA (1∼50 ng) was varied so that PCR products were obtained during the log phase of the PCR amplification.

### Preparation of photoreceptor outer segments (POS)

POS were isolated according to established protocols from bovine eyes obtained fresh from the slaughterhouse [19]. POS were stored suspended in 10 mM sodium phosphate, pH 7.2, 0.1 M sodium chloride, 2.5% sucrose at −80°C. Before use, POS were thawed and labeled by addition of 20% vol of 1 mg/ml FITC (Molecular Probes) in 0.1 M sodium bicarbonate, pH 9.0, for 1 hr at room temperature in the dark. POS were then washed and re-suspended in cell culture media.

### Phagocytic activity assay

The phagocytic activity assay was done based on a published method [20]. Briefly, isolated POS aliquots were stored at −80°C at a concentration of 10^9^ POS/mL. Before use, POS was labeled with 1 mg/mL fluorescein isothiocyanate (FITC) (Molecular Probes, Carlsbad, CA) in 0.1 M Na-bicarbonate (pH 9.0), for 1 hr in the dark, before being washed and resuspended in cell culture medium with 2.5% sucrose. The RPE cells were fed with FITC-POS (10 POS/RPE cell) for 3 hrs under culture conditions before rinsing four times with PBS containing 1 mM MgCl_2_ and 0.2 mM CaCl_2_. The total fluorescence was recorded at 485/525 nm using Tecan plate. Each assay was repeated four times. Intensities were calculated with Graph Pad Prism.

### Cathepsin D enzyme activity in vitro

Cathepsin D activity was measured in ARPE-19 cell extracts using a kit containing a fluorogenic peptide substrate peptide, MOCAc-Gly-Lys-Pro-Ile-Leu-Phe-Phe-Arg-Leu-Lys(DNP)-D-Arg-NH2 (Sigma CS0800), reaction buffer (pH 4.0), and standards. Reactions were initiated by the addition of substrate and kinetics of substrate hydrolysis was measured using a fluorescent plate reader (Biotek Synergy2 Ex 340 nm, Em 460 nm) at 37°C for 15 min with data points collected every 120 sec. Data was imported to Graph Pad Prism for analysis and determination of initial rates, and normalization to total protein assayed.

### Cathepsin D enzyme activity in vivo

The targeted peptide substrate (R9-CatD) was developed by Fischer and coworkers [21]. Peptide R9-CatD contains an N-terminal nona-arginine tract (R9) that allows uptake by cells and localization in the lysosomes. The remainder of the substrate, Lys(Rhod)-Ala-Pro-Ile-Ser-Phe-Phe-Glu-Leu-Lys(Fluor), has two fluorophores. A fluoroscein (Fluor) is at the C-terminus and a tetramethylrhodamine (Rhod) is at the N-terminus of the cathepsin D cleavage sequence. Because of the overlap of the emission and the excitation spectrum of the dyes, the intact peptide has quenched fluoroscein emission. Fluoroscein fluorescence increases with substrate cleavage because of the dissociation of the C-terminal fluoroscein fluorophore peptide from the remainder of the substrate. The fluorescence ratio Fluor/Rhod allows the degree of cleavage to be determined in a substrate concentration independent manner. After the desired treatment with agents, cells were washed with serum and dye free cell culture media (DMEM F12 with HEPES) containing R9-CatD peptide at 2 µM for 1 hr in serum free media. The media was removed and cells rapidly washed twice with PBS. Cells were harvested by adding lysis buffer (200 µL, Cytobuster) and incubated at room temperature for 30 min. Then fluoroscein (485/525) and rhodamine fluorescence (580/620) were sequentially recorded using a fluorescence plate reader.

### Exosome isolation

All procedures were done at 4°C. ARPE-19 cell culture media was sequentially purified by removing the pellet after centrifugation for 10 min at 300×*g*, 10 min at 2000×*g*, 30 min at 10,000×*g* and 1 hr at 100,000×*g*. The final supernatant was removed as completely as possible and the pellet re-suspended in 1 ml PBS. The re-suspended pellets were pooled from all the tubes containing materials from the same cells into a single centrifuge tube. Then PBS was added to fill the tube and the tube was ultracentrifuged at 100,000×*g* for 1 hr. The supernatant above the visible pellet was removed and the exosome preparations were re-suspended in 100–200 µl of fresh PBS.

### Analysis of exosomes by flow cytometry

Exosomes were measured using flow cytometry (FACS) to quantify the levels of proteins on the outer membrane [22]. Briefly, exosomes were fixed to beads (4.0 µm latex beads, 4% solids, Interfacial Dynamics 12–4000) by incubation of 5 µg purified exosomes, as measured by Bradford assay, with 10 µl latex beads for 15 min at room temperature in a 1.5 ml microcentrifuge tube. Then PBS was added to a final volume of 1 ml and the preparation was incubated on a test tube rotator wheel for 2 hr at room temperature. 110 µl of 1 M glycine was added (i.e., 100 mM final), mixed gently and let stand on the bench at room temperature for 30 min. Beads were re-suspended in 0.5 ml PBS/0.5% BSA, 10 µl coated beads were incubated with 50 µl primary antibodies: mouse anti-CFH (1∶50, Serotec), mouse anti-CD63 (1∶50, Abcam), mouse ant-LAMP2 (1∶50, Abcam), mouse anti-CD81 (1∶50, Abcam), mouse anti-C3 (1∶50, Abcam). Primary antibody diluted in PBS/0.5% BSA for 30 min at 4°C and then washed twice with 150 µl PBS/0.5% BSA. The beads were then incubated with 50 µl anti-mouse Alexis red (1∶50, Molecular Probes) secondary antibody diluted in PBS/0.5% BSA for 30 min at 4°C, washed twice in PBS/BSA, and re-suspended in 500 µl of PBS/BSA. Antibody-labeled exosome-coated beads were analyzed on a flow cytometer (DakoCytomation CyAn) with the forward scatter (FSC) and side scatter (SSC) adjusted to see both single beads and bead doublets. Comparisons were made between fluorescence obtained with specific antibody and with irrelevant isotype controls.

### CFH/exosome binding

A series of concentrations of CFH: 0, 0.5, 0.05, 0.005, 0.0005 mg/mL were incubated with exosomes overnight at 4°C, washed in PBS/BSA, and then incubated with 50 µl mouse anti-C3 (1∶50, Abcam) or mouse anti-CFH (1∶50, Serotec) primary antibody diluted in PBS/0.5% BSA for 30 min at 4°C. The preparation was washed twice with 150 µl PBS/0.5% BSA, incubated with 50 µl secondary antibody diluted in PBS/0.5% BSA for 30 min at 4°C, washed twice in PBS/BSA and re-suspended in 500 µl of PBS/BSA. The antibody-labeled exosome-coated beads were analyzed in a DakoCytomation CyAn flow cytometer.

### Release of cytokines

Human cytokine array Panel A (R & D) was used for the determination of cytokines released from ARPE-19 cells after mtDNA damage (as described above). Briefly, cells were seeded at a density of 50,000 cells/well into 24-well plates. One day after seeding, the plates were treated with media containing 0 or 2.5 µM rotenone. After the cells were treated for 24 hr, the supernatant was collected and the cytokine profile was determined, following the manufacturer's instructions.

### MCP-1 and MIF ELISA

The release of selected cytokines from ARPE-19 cells was quantitatively determined after exposure to rotenone at concentrations of 0, 0.3, 0.6, 1.2 or 2.5 µM. Culture medium samples were collected after 24 hr, centrifuged and the supernatant in each group was used to measure the levels of MCP-1 and MIF with an ELISA kit, following the manufacturer's instructions (R&D Systems, MN).

## Results

### Autophagy marker, Atg5, in drusen

**Figure 1:**
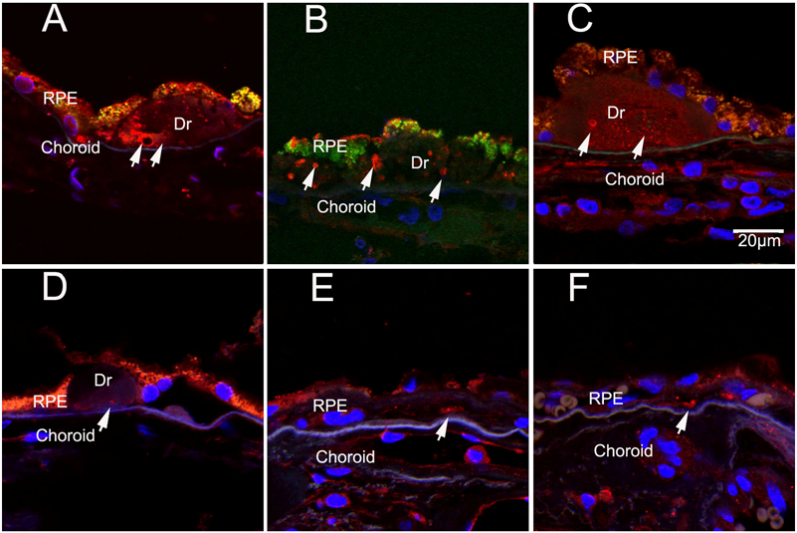
Immunolocalization of autophagy marker, Atg5, in the human RPE/choroid. In these confocal immunofluorescence images: an-anti-Atg5 antibody labels particles in drusen (arrows). (A) AMD eye from 94-year-old male; (B) AMD eye from 97-year-old male; (C) AMD eye from 74-year-old male; (D) non-AMD eye from 75-year-old male ; (E) non-AMD eye from 60-year-old male; (F) non-AMD eye from 87-year-old male. Dr, drusen. Scale bar = 20 µm. Blue: DAPI; red: Atg-5; Green: autofluorescence.

We found that Atg5, an autophagy marker, was present in drusen in the retina of normal old eyes and old eyes with AMD. The distribution of Atg5 was assessed in the eyes of ten donors (twelve eyes) with AMD and six donors of similar age (twelve eyes) without AMD (representative samples are shown in Fig. 1). In donors with AMD, a cluster of Atg5 immunoreactivity was present in the large drusen found in the sub-RPE space (Fig. 1 A–C, three different donors). In age-matched eyes without AMD, Atg5 was present as small isolated spots in drusen and the sub-RPE space (D–F, three different donors).

### Increased autophagy markers in the aged mouse RPE/choroid

**Figure 2:**
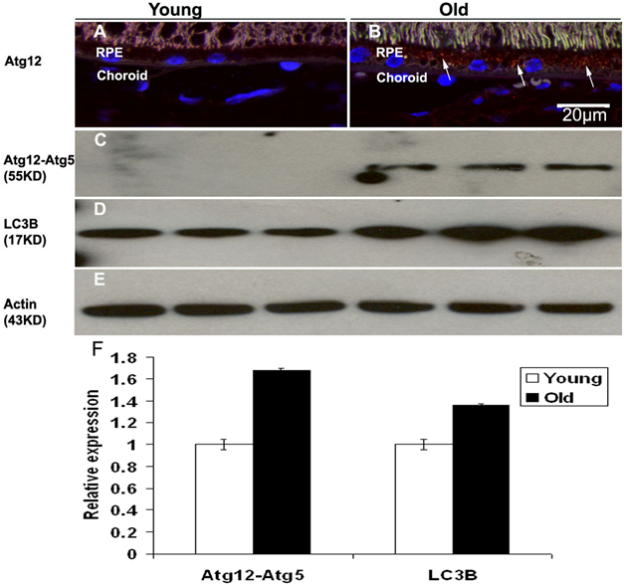
Increased autophagy markers in old mice. (A–B): Localization of Atg12 in young and old RPE/choroid in the mouse eye. (A) In the eyes from young mice, there was no Atg12 labeling (red) in the RPE/choroid tissue. (B) In the RPE/choroids from old eyes, there was spotted labeling (red, arrows) in the RPE. Scale bar = 20 µm. (C–E): Comparison of protein levels of (C) Atg12-Atg5 conjugates and (D) LC3B in RPE/choroid of young and old mice by immunoblot. Atg12-Atg5 conjugates and LC3B were all increased in RPE/choroid from old animals. (E) β-actin was used as a loading control. (F): The differences in expression levels were determined by multiple scans of blots to ensure a maximium and minimum response range for the measured areas and the integrated areas of the bands were calculated by using Image-J software. Data were expressed as normalized ratios (Young  = 1). There were significant increases in aged retinas of Atg12-Atg5 (p<0.05, n = 3) and LC3B (p<0.05, n = 3), compared to young retinas. Values are the mean±SEM. Appropriate background subtraction and normalization of the data to actin was done for each blot.

In mammalian cells, an Atg12-Atg5 conjugate associates with the membranes of precursor autophagosomes [23]. We examined the autophagy conjugation system by detecting Atg12-Atg5 using a specific monoclonal antibody raised against a full-length recombinant Atg12. There was immunolabeling for ATG12 in the RPE of old mice but no immunolabeling in the RPE of young mice (Fig. 2A and B). By Western blots (Fig 2C–F), we found that autophagy markers, Atg12-Atg5 and LC3B, were increased in RPE/choroids in old mice (24–28 mos) compared to young mice (4 mos).

### In vitro autophagy induction in an RPE cell line by rotenone treatment

Autophagy can be initiated by accumulation of damaged macromolecules, for example damaged mtDNA, during aging [24]. To study the autophagy process that occurs in response to damage of mtDNA, we established an in vitro autophagy model system using rotenone, a widely used inhibitor of mitochondrial complex I. Our strategy was to use rotenone at a low concentration so that the respiratory activity of the mitochondria was not substantially inhibited but ROS were generated that could damage mtDNA.

**Figure 3:**
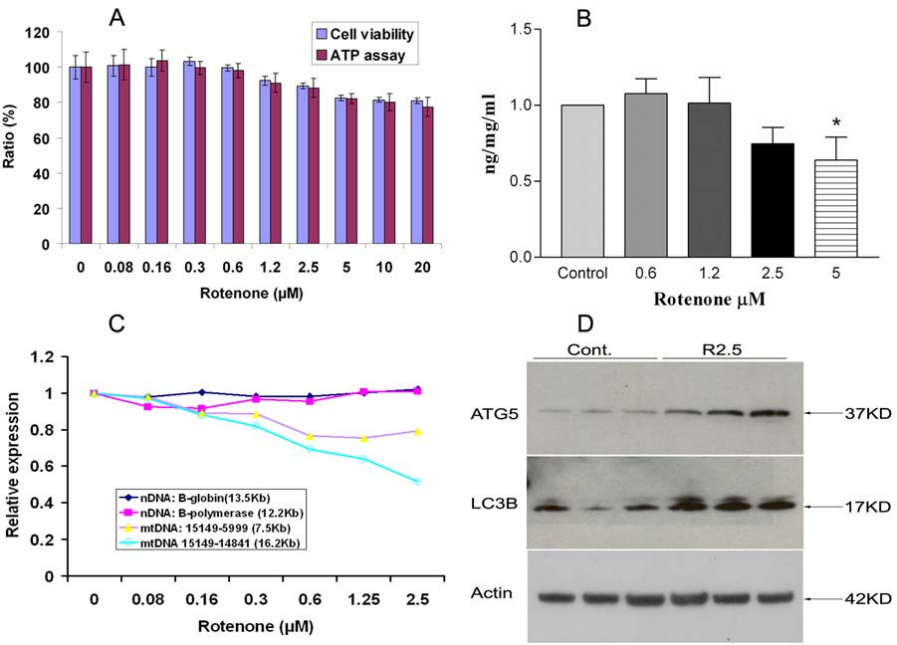
Induction of autophagy in ARPE-19 cells. (A): Cell viability assay and ATP assay for ARPE-19 cells treated with rotenone. Concentrations in the media of 0.08 to 2.5 µM did not cause a significant decrease of cell viability or ATP levels. (B): Rotenone at concentrations in the media of 0.6 to 2.5 µM did not cause a significant decrease in CoQ levels. (C): Rotenone at concentrations in the media of 0.6 to 2.5 µM damaged mtDNA, but not nDNA. (D): Comparison of protein levels of Atg12-Atg5 conjugates and LC3B in ARPE-19 cells by immunoblots. Atg12-Atg5 conjugates and LC3B were increased in ARPE-19 cells after 2.5 µM rotenone treatment. β-actin was used as a loading control.

ARPE-19 cells are a transformed human RPE cell line, widely used for studying the RPE in vitro. We determined the concentrations of rotenone that would not affect ARPE-19 cell viability as measured by the MTT assay (Fig. 3A). Although ROS were produced (data not shown), rotenone at concentrations 0.08 µM∼2.5 µM did not significantly affect cell viability. To confirm the lack of impact of low concentrations of rotenone on overall mitochondrial activity, ATP levels were measured using the ATP luminescence assay. Fig. 3A demonstrates that rotenone at concentrations 0.08 µM∼2.5 µM did not significantly affect the cellular ATP level. Student's t test and one way ANOVA were performed to determine if there were significant changes in cell viability and ATP levels with and without rotenone treatment. To further confirm that low concentrations (0.6–2.5 µM) of rotenone did not inhibit mitochondrial respiratory activity, we determined that these concentrations of rotenone did not affect the product of mitochondrial complex I, CoQ. As shown in Fig. 3B, 0.6 µM∼2.5 µM rotenone did not affect the level of CoQ.

### Quantification of damaged mtDNA and nDNA in cultured RPE

Having established that rotenone at low concentrations (0.08 µM∼2.5 µM) had a minor effect on viability, ATP and CoQ, we then determined whether this treatment damages the mtDNA and/or nuclear (nDNA). Genomic DNA from six independent preparations in each group was isolated with DNeasy Blood & Tissue Kit (Qiagen). Using LX PCR, there was a significant decrease of the relative amplification of mtDNA, but not nDNA (Fig. 3C), indicating damaged mtDNA in the concentration range of 0.6 µM–2.5 µM rotenone, compared with no rotenone treatment (*p*<0.05, n = 6). Thus, low concentrations of rotenone used in vitro in an RPE cell line caused, at least in part, a characteristic of the aging cell, damaged mtDNA. Agarose gel electrophoresis showed that all mtDNA PCR products were single bands of the appropriate size (Figure S1). Data are presented as mean±(SEM) with statistical differences between groups analyzed by standard two-tailed t-test using GraphPad Prism software. A *p* value of less than 0.05 was considered statistically significant.

### Increased autophagy markers associated with mtDNA damage

We measured autophagy markers, Atg5 and LC3B, using Western blot in ARPE-19 cells that had damaged mtDNA caused by rotenone treatment. As shown in Fig. 3D, Atg12-Atg5 conjugates and LC3B were increased ARPE-19 cells with mtDNA. Quantitation of Atg5 and LC3B is shown in Supplementary Figure S2. We interpret these results to indicate that there was increased autophagy after damage to mtDNA, another characteristic of the aging cell.

### Phagocytosis

**Figure 4:**
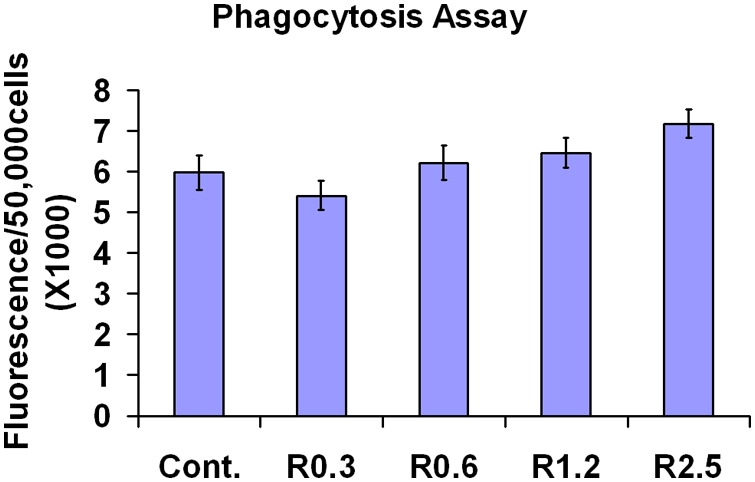
Phagocytotic activity of ARPE-19 cells. Rotenone at concentrations in the media of 0.3–2.5 µM did not change phagocytic activity when cells were exposed to photoreceptor outer segments for 3 hr (p<0.05, n = 4).

Functionally, RPE cells are among the most active phagocytic cells in the body [25]. To determine whether mtDNA damage and autophagy affects phagocytosis, we measured the phagocytosis of fluorescence labeled POS by ARPE-19 cells (Fig. 4). When cells were exposed to fluorescence labeled POS for 3 hr, there was no difference in the fluorescence intensity at different concentrations of rotenone. The total fluorescence intensities from four independent preparations in each group were calculated. Data are presented as mean±SEM with statistical differences between groups analyzed by standard two-tailed t-test using GraphPad Prism software. A p value of less than 0.05 was considered statistically significant. The damage to mtDNA and the increased autophagy that we had caused in ARPE-19 cells had no impact on phagocytic activity.

### Lysosomal activity

**Figure 5:**
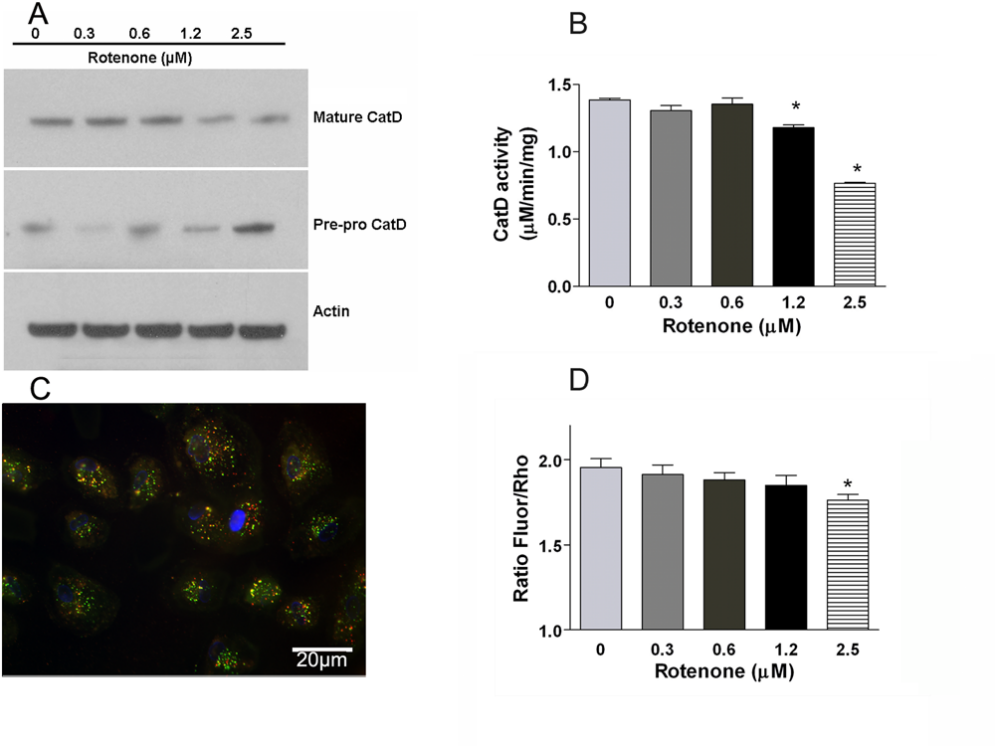
Lysosomal activity. (A) Comparison of protein levels of mature cathepsin D (33kD) and pre-pro-cathepsin D (48–52 kD) in ARPE-19 cells by immunoblot. β-actin was used as a loading control. (B) Cathepsin D enzymatic activity from cell extracts were significantly decreased at 1.25–2.5 µM concentrations of rotenone (*p*<0.05). (C) immunofluorescence of *in vivo* cathepsin D substrate showing uptake into the lysosomes of ARPE-19 cells (red: total substrate; green: cleaved substrate; blue: DAPI). (D) *In vivo* lysosomal activity assay showing lysosomal activity against a peptide substrate for cathepsin D was significantly decreased at 2.5 µM rotenone (*p*<0.05). Scale bar = 20 µm.

Another major function of the RPE is to digest damaged intracellular macromolecules arising from endosomes, phagosomes and autophagy by the fusion with lysosomes. We determined whether damage to mtDNA and increased autophagy affected lysosomal activity. The most important lysosomal enzyme in RPE cells is the aspartic protease cathepsin D [26]. Cathepsin D protein levels were significantly decreased after mtDNA damage at 1.25–2.5 µM rotenone, as demonstrated in the western blots in Fig. 5A. Quantification of cathepsin D protein levels is shown in Supplementary Figure S3. We used in vitro and in vivo assays to measure cathepsin D activity. Cathepsin D enzymatic activity from cell extracts was significantly decreased at 1.25–2.5 µM rotenone (Fig. 5B). Five independent preparations in each group were used for statistical analyses. Data are presented as mean±SEM with statistical differences between groups analyzed by standard two-tailed t-test using GraphPad Prism software. A p value of less than 0.05 was considered statistically significant. In addition, we used an in vivo cathepsin D activity assay to observe the impact of mtDNA damage, induced by rotenone treatment, and increased autophagy on cathepsin D activity (Fig. 5C and D). Intracellular cathepsin D activity was significantly decreased at 2.5 µM rotenone. The change in enzyme activity is consistent with the change in protein levels (Fig. 5A).The results in Fig. 5, using western blot and two different measures of cathepsin D activity, independently confirmed that damage to mtDNA in ARPE-19 cells was associated with a small but significant decrease in cathepsin D activity. Interestingly, using Western blots we found that pre-pro-cathepsin D protein was increased at low concentrations of rotenone treatment, which suggests that there was less processing of pre-pro-cathepsin D to the mature form (Fig. 5A). The mechanism by which mtDNA damage induced by rotenone treatment alters the level of cathepsin D in the RPE needs to be elucidated.

### Exocytotic activity in stressed RPE

We reasoned that when RPE cells with damaged mtDNA, increased autophagy and decreased lysosomal activity are presented with POS, additional mechanisms must be needed to handle the damaged intracellular macromolecules. We hypothesized that compromised RPE will remove damaged intracellular macromolecules without completely digesting them by increasing exocytotic activity. Endosomes and the exosomes that they form and release are capable of removing intracellular macromolecules.

**Figure 6:**
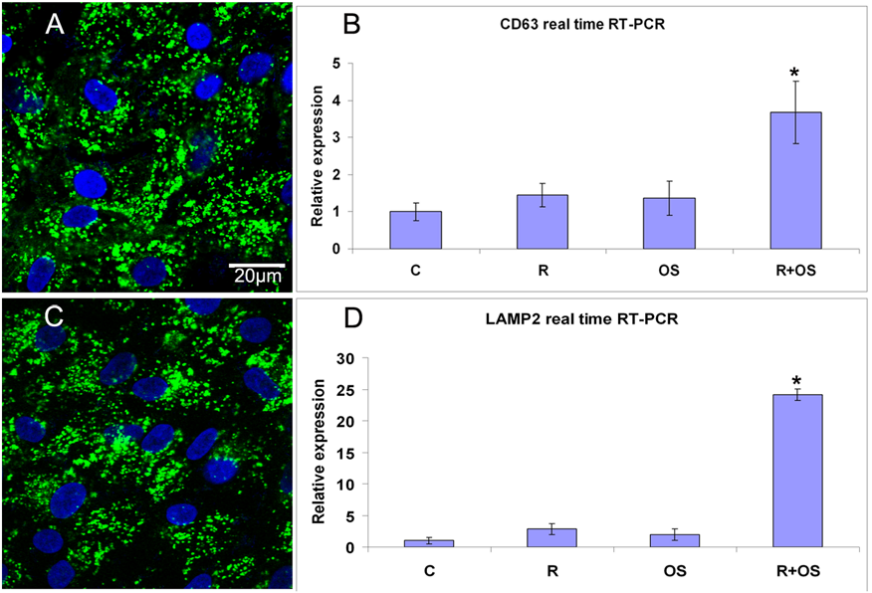
Exosome markers in ARPE-19 cells. (A) Localization of CD63 in ARPE-19 cells without treatment. (B) 2.5 µM rotenone for 24 hrs or exposure to POS 12 hrs alone did not change CD63 expression at 36 hrs. However, when the cells were treated with rotenone for 24 hrs to damage their mtDNA and then fed POS for 12 hrs, CD63 was significantly increased (*p*<0.05). (C) Localization of LAMP2 in ARPE-19 cells without treatment. (D) Exposure to 2.5 µM rotenone for 24 hrs alone or POS for 12 hrs alone did not change LAMP2 expression. However, when the cells were treated with rotenone for 24 hrs to damage their mtDNA and then fed POS for 12 hrs, LAMP2 was significantly increased at 36 hrs (*p*<0.05).

We measured two markers for late endosomes and exosomes: CD63 and LAMP2. As shown in Fig. 6A–D, exposure to 2.5 µM rotenone for 24 hrs alone or exposure to POS for 12 hrs alone did not change CD63 and LAMP2 gene expression at 36 hrs. However, when the cells were treated with rotenone for 24 hrs, and then exposed to POS for 12 hrs to increase phagocytosis, expression of CD63 and LAMP2 were significantly increased. We speculate that as a result of mtDNA damage and decreased lysosomal activity, the cells increase their exocytotic activity as a means to eliminate intracellular debris. The autophagy markers that appear in drusen in the retina with age (Fig. 1) may be due, in part, to an age-related cumulative increase of exocytotic activity.

### Exosome markers in aged mouse RPE/choroids

**Figure 7:**
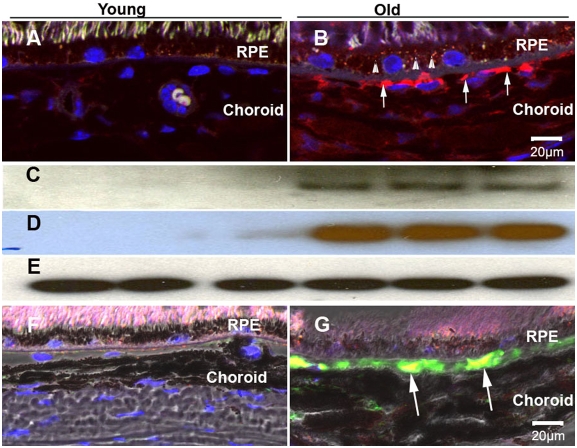
Exosome markers in RPE/choroid of mice. (A–B): Localization of CD63 in young and old RPE/choroid. (A) In the young RPE/choroid, there was no CD63 labeling in the RPE layer or Bruch's membrane. (B) In the old RPE/choroids, there was labeling in the RPE layer in old animals (small arrow heads). CD63 deposition at Bruch's membrane showed large and discontinuous clumps (arrows). (C–E): Comparison of protein levels of CD63 and LAMP2 in RPE/choroid of young and old mice by immunoblot. (C) CD63 and (D) LAMP2 were increased in the RPE/choroid from old animals. (E) β-actin was used as a loading control. (F–G): Co-localization of CD63 (green) and C3 (red) in young and old RPE/choroids. (F) In the young RPE/choroid, there was no co-localization of CD63 and C3 in the RPE layer or Bruch's membrane. (G) In the old RPE/choroid, C3 co-localized with CD63 at Bruch's membrane (arrows). Figure F and G were overlay images with the brightfield image. Scale bar = 20 µm.

We then asked whether we could find evidence for increased exocytotic activity in the RPE/choroid with age. Six mouse eyes were used for each group in immunohistochemistry experiments and three mouse eyes were used for each group in Western blots. Using immunohistochemistry and Western blots, we found exosome marker proteins, CD63 and LAMP2 between RPE and choroid in old, but not in young, mouse tissue (Fig. 7A–B). Western blots of RPE/choroid from young and old eyes confirmed the marked increase of CD63 and LAMP2 in old tissue (Fig. 7C–E). C3 is a component of drusen [3] and C3 polymorphisms are associated with AMD [27]. Our laboratory previously demonstrated that C3 increases with age and in the aged RPE/choroid C3 deposits are large and discontinuous clumps along Bruch's membrane [28]. Interestingly, CD63 co-localized with C3, suggesting an extracellular interaction between exosome proteins and complement (Fig. 7 F–G). Thus, with age there is an accumulation of exocytotic markers in the region of Bruch's membrane in mice.

### Exosomes contain proteins found in human drusen

**Figure 8:**
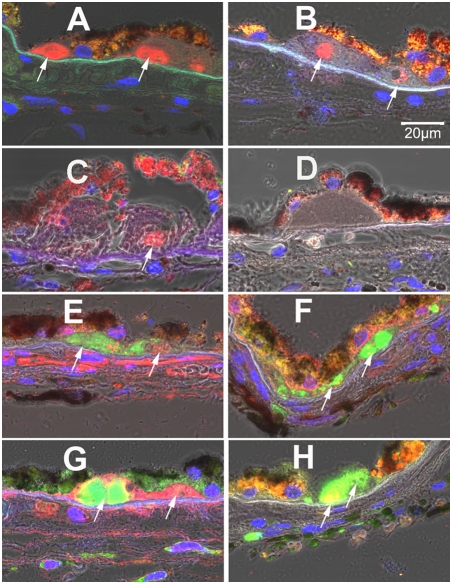
Immunolocalization of exosome markers in the RPE/choroid complex. (A): CD63 (red); AMD eye from a 74-year-old male. Anti-CD63 antibody labels large amorphous areas (CD63: arrows) in drusen. (B):CD81 (red); AMD eye from a 96-year-old male (CD81, arrows). (C): LAMP2 (red). AMD eye from a 93-year-old (LAMP2, arrow). (D): CD63 (red); Non-AMD eye from a 75-year-old male as an age-matched control. No CD63 labeling was seen in the drusen from any age-matched control (n = 10). (E–H): CD63 (green) co-localization with proteins that are known to be in drusen. (E) amyloid β (red) showed no co-localization with CD63 (arrow). (F) α B crystalline (red) showed co-localization with CD63 (arrowhead). (G) C5b-9 (red) showed no co-localization with CD63 (arrow). (H) CFH (red) showed co-localization with CD63 (arrowhead). Dr, drusen. Blue: DAPI.

We found that exosome markers, CD63, CD81, LAMP2, were present in drusen from AMD patients (Fig. 8A–C, three different donors), but not in age-matched controls (Fig. 8D). Furthermore, CD63 co-localized with proteins that are well known in human drusen: amyloid β, αB-crystalline, C5b-9 and CFH (Fig. 8E–H).

### CFH binding to exosomes

**Figure 9:**
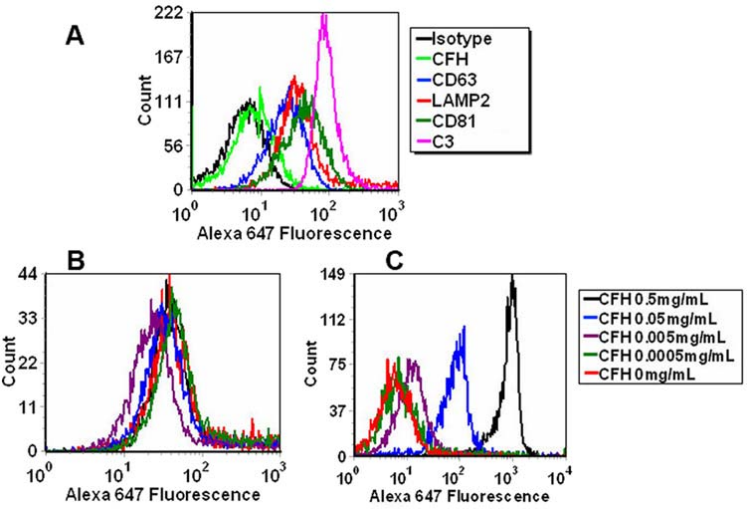
Analyses of exosomes by FACS. (A) Exoxomes are CD63, LAMP2, CD81 and C3 positive, but CFH negative. Isotype control: Black; CFH: light green; CD63: blue; LAMP2: red; CD81: dark green; C3: magenta. (B) Added CFH bound to exosomes in a dose-dependent manner. (C) Added CFH did not alter the amount of C3 on exosomes. For B and C: CFH 0.5 mg/mL: black; CFH 0.05 mg/mL: blue; CFH 0.005 mg/mL: purple; CFH 0.0005 mg/mL: green; CFH 0 mg/mL: red.

We reasoned that released exosomes might be a target of the complement system. We therefore determined whether exosomes from ARPE-19 cells bind C3 and/or CFH and if CFH binding affected the amount of C3 associated with the exosomes. To verify that we could collect exosomes released from RPE, we collected exosomes from the cell media of ARPE-19 cells and demonstrated the markers of exosomes. Using FACS, we found that exosomes were, as expected, CD63, CD81 and LAMP2 positive (Fig. 9A: blue, dark green and red lines respectively). Interestingly, these exosomes were C3 positive (magenta line) but CFH negative (light green line) (Fig. 9A). As shown in Fig. 9B, exosomes bound CFH in a dose dependent manner. Binding of CFH did not alter the amount of C3 (magenta line) associated with exosomes (Fig. 9C). Thus, C3 coated exosomes released from the RPE, are possible targets for CFH.

### Cytokine and chemokine profiles

Our previous data, comparing gene expression profiles from normal, young and old mice, show marked upregulation of genes that are responsible for leukocyte extravasation in the aged RPE/choroid. Prominent in the aged retina was the expression of macrophage chemoattractant protein-1 (MCP-1), a chemokine that attracts macrophages. We hypothesized that RPE cells with decreased lysosomal activity and increased exocytotic activity will release signals for recruitment of leukocytes, particularly macrophages, to aid in the digestion of extracellular deposits.

**Figure 10:**
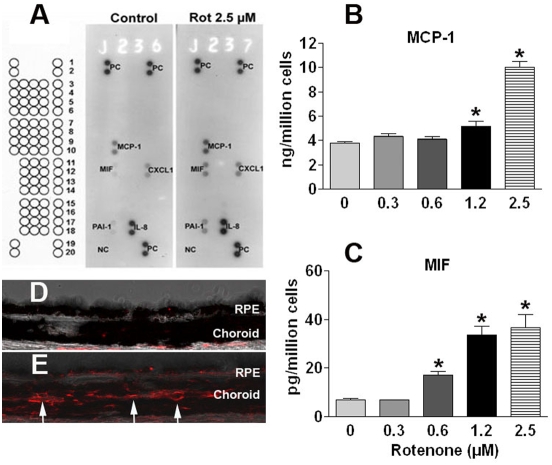
Cytokines secreted from RPE cells and macrophage infiltration into old mouse RPE. (A) Human Cytokine Array of media from ARPE-19 cells. MCP-1, MIF, CXCL1, IL-8 and PAI-1 were detectable in both the control group and after rotenone treatmen t and appear to increase under conditions of increased mtDNA damage. (B) MCP-1 ELISA. MCP-1 protein from cell media was significantly increased at 1.25–2.5 µM concentrations of rotenone (*p*<0.05). (C) MIF ELISA. MIF protein from cell media was significantly increased at 0.6–2.5 µM concentrations of rotenone (*p*<0.05). (D) Absence of F4/80 labeled macrophages in young RPE/choroid tissue (overlaid with a brightfield image; (E) F4/80 labeled macrophages infiltrated into old RPE/choroid tissue (red). Arrows indicate that most of the macrophages are on the choroid side of Bruch's membrane.

To determine whether stressed RPE due to damaged mtDNA and increased autophagy produce signals to recruit leukocytes, we used the Human Cytokine Array for parallel determinations of 36 cytokines and chemokines (Fig. 10A). ARPE-19 cells treated with rotenone (2.5 uM, 24 hr) exhibited increased release of MCP-1 and migration inhibitory factor (MIF). We confirmed these changes quantitatively by ELISA (Fig. 10B–C). These results predicted increased macrophages in eyes from old animals. In vivo in old mice eyes, we found infiltration of F4/80 positive macrophages into the RPE/choroid (Fig. 10D–E). These results are consistent with the recruitment of leukocytes into the aged RPE in rodents and the increased immunological activity with age [28].

## Discussion

The autophagy activity of living cells increases with age and is due to an increased intracellular burden of damaged macromolecules and organelles caused by reactive oxygen species during aging [29]. Mitochondria are targets for oxidative stress from the generation of free radicals which cause damage to the mtDNA. Mitochondria with damaged mtDNA fuse with lysosomes for degradation via a form of autophagy known as macroautophagy [7]. Using an in vitro RPE model, we found that autophagy is associated with mtDNA damage. Autophagy may exist in vivo in the RPE of old eyes. We have demonstrated that there are markers for autophagy in the RPE/choroid of old mice. We have also found that autophagy markers are present in drusen in the human retina. We provide the first evidence for increased autophagy activity in the aging RPE and the presence of autophagy markers in drusen of the eyes of old human donors. An in-depth study of autophagy in the aging RPE and how markers of autophagy become a component of drusen is warranted.

A major function of the RPE is the phagocytosis and digestion of photoreceptor outer segments. Our results indicate that RPE cells with mtDNA damage are competent in the internalization steps of phagocytosis. Digestion of POS requires cathepsin D proteolytic activity in the lysosome [26]. We found a significant decrease in cathepsin D activity in cultured ARPE-19 cells. However, increased cathepsin D activity has been reported for the human RPE with age [30].Thus, this result of in vitro modeling of mtDNA damage in ARPE-19, which is a human, transformed cell line, are not consistent with the in vivo findings. Perhaps the RPE in an old eye in vivo has the ability to upregulate cathepsin D activity as a response to age-related, intracellular accumulation of damaged molecules and organelles. Whether the aged RPE can process the age-related mtDNA damage, increase autophagy activity and maintain the daily function of phagocytosis of POS remains to be determined.

Intracellular overload of damaged macromolecules leads to increased exocytotic activity. Exocytotic activity includes the formation of endosomes, multi-vesicular bodies and the release of exosomes from the cell. Released exosomes carry undigested or partially digested intracellular proteins out of the cell. Although it is not possible to collect exosomes from a tissue in vivo, our model of autophagy in ARPE-19 cells demonstrates that exosome markers are upregulated when the cells are stressed by oxidative damage to mtDNA and are presented with POS to digest. In vivo, the RPE in the aged eye may be releasing increased numbers of exosomes containing intracellular proteins. We provide the first evidence for an apparent increased exocytotic activity in the aging RPE. Interestingly, there are increased exosome markers surrounding Bruch's membrane in the old mouse eye, implying that released exosomes or their contents are being trapped locally in the tissue. Consistent with the old mouse eye, there are exosome markers in drusen from eyes of AMD patients. We postulate that age-related, increased release of exosomes externalizes intracellular proteins.

Based on published studies of exosome proteomics from different cell types, we found marked similarity of the intracellular proteins reported in the literature that are found in drusen [3], [31] and the intracellular proteins that are found in exosomes [32] (Table 2). Furthermore, recent proteomic analyses of Bruch's membranes from AMD patients have detected over 80 proteins that are reported to be excreted in exosomes, including the exosome markers: CD63, CD81 and CD9 (personal communication, John W Crabb). Therefore, the exocytosis process may contribute intracellular proteins which become building material for the formation of drusen on Bruch's membrane.. Certainly, an in-depth study of the exocytotic activity in the RPE of AMD patients is likely to be fruitful.

**Table 2:** Comparison, from the literature, of exosome proteome and drusen proteome

	Drusen [3], [31]	Exosomes [32]
**Nuclear**	X	X
histone, H1	X	X
histone, H2A	X	X
histone, H2B	X	X
histone, H3	X	X
histone, H4	X	X
**Mitochondria and cytoplasm**		
aldolase A	X	X
enolase 2	X	X
lactate dehydrogenase A	X	X
pyruvate kinase, M1 isozyme	X	X
malate dehydrogenase 1	X	X
phosphoglycerate kinase 1	X	X
14-3-3 β	X	X
apolipoprotein A1	X	X
phospholipase A2	X	X
polyubiquitin	X	X
ubiquitin	X	X
peroxiredoxin	X	X
triosephosphate isomerase	X	X
myosin	X	X
**Cytoskeleton**		
actin, β	X	X
actinin, α	X	X
tubulin α and β	X	X
vimentin	X	X
**Cell membrane**		
annexin I	X	X
annexin II	X	X
annexin V	X	X
annexin VI	X	X
**Serum**		
complement C3	X	X
albumin	X	X
hemoglobin	X	X
ceruloplasmin	X	X
plasminogen	X	X

We hypothesize that, in the aged RPE in vivo, there is age-related, increased exocytotic activity lead to the release of intracellular proteins via exosomes which contribute to the formation of drusen. Blood proteins and extracellular proteins are normally present in the RPE/choroid extracellular environment. We suggest that in the aging eye the formation of drusen is initiated by intracellular proteins from the RPE that become extracellular via exosomes.

Although we have not studied any one process in detail, our findings link aspects of aging, oxidative stress, mtDNA damage, autophagy, lysosomal activity, exocytotic activity as functional processes of RPE that have changed in the aged eye. Clearly, age-related changes in autophagy, exocytosis and other functions of the RPE need to be studied further. These altered cell biological processes in the old eye may underlie susceptibility to genetic mutations that are found in AMD patients and may be associated with the pathogenesis of AMD in the elderly.

Intracellular overload of damaged macromolecules may lead to inflammatory cells entering the aged RPE/choroid. For example, macrophages may invade the tissue in response to increased expression of MCP-I and MIF, which we demonstrated occurs after mtDNA damage. In previous work, we demonstrated that the aged RPE/choroid becomes immunologically active [28]. Human genetics investigations have focused on the CFH gene, a mutation of which increases risk of AMD in both homozygotes and heterozygotes [33], [34]. Our observation that exosomes released by ARPE-19 cells are coated with C3 suggests that exosomes interact with the complement pathways. C3 coated exosomes from the RPE which, as we have shown, bind CFH may be stabilized and removed by the circulation. A mutation in *CFH* may make C3 coated exosomes, released from RPE, targets for the immunological activity of the invading leukocytes in the old RPE/choroid. In the presence of invading leukocytes in the aged tissue, CFH dysfunction in the complement pathway may cause destabilization of the exosome membrane release of intracellular proteins into the external environment and, possibly incorporation of intracellular proteins into drusen. These events, to the extent that they occur in the human macula, may lead to the pathogenesis of AMD.

## Supporting Information

Figure S1 Agarose gel electrophoresis of mtDNA PCR products. All were single bands of the appropriate size. Lane 1: {lower case lambda DNA-HindIII digest standards; Lane 2: empty; Lane 3: 16.2 Kb mtDNA (arrow), control; Lane 4: 16.2 Kb mtDNA (arrow), 2.5 µM rotenone treatment; Lane 5: 7.5 Kb mtDNA (arrow), control; Lane 6: 7.5 Kb mtDNA (arrow), 2.5 µM rotenone treatment.
(0.10 MB TIF)

Figure S2 Expression of Atg5 and LC3B in ARPE-19 cells. The differences in expression levels were determined by multiple scans of blots to ensure a maximium and minimum response range for the measured areas and the integrated areas of the bands were calculated by using Image-J software. Data are expressed as normalized ratios to actin. Values are the mean±SEM. Appropriate background subtraction and normalization of the data to actin was done for each blot. There were significant increases in 2.5 µM rotenone treatment of Atg5 (p<0.05, n = 3) and LC3B (p<0.05, n = 3), compared to normal controls.
(0.13 MB TIF)

Figure S3 Expression of cathepsin D in ARPE-19 cells. The differences in expression levels of cathepsin D were determined by scanning gels and determining the integrated areas of the bands using Image-J software. Data are expressed as normalized ratios to actin. Values are the mean±SEM. Appropriate background subtraction and normalization of the data to actin was done for each blot. There were significant decreases in 1.25 and 2.5 µM rotenone treatment of cathepsin D (p<0.05, n = 5), compared to normal controls.
(2.93 MB TIF)
