# Supplementary figures and images for: Autophagy and Exosomes in the Aged Retinal Pigment Epithelium: Possible Relevance to Drusen Formation and Age-Related Macular Degeneration
Source: PLoS One. 2009 Jan 8;4(1):e4160. doi: 10.1371/journal.pone.0004160 (PMC2612751; doi:10.1371/journal.pone.0004160)

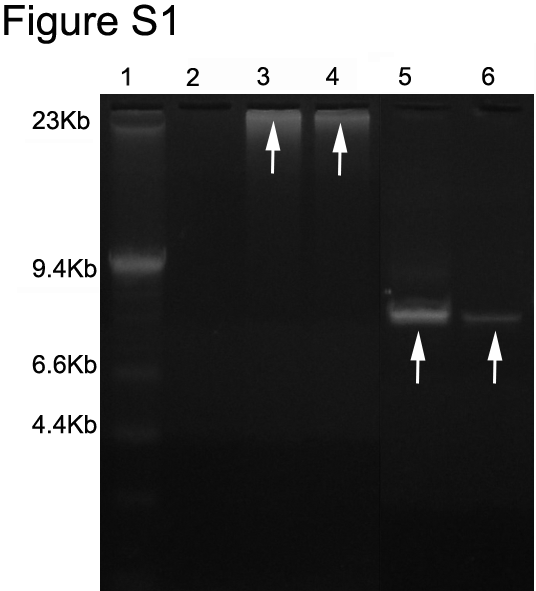

Supplement: Figure S1 — Agarose gel electrophoresis of mtDNA PCR products. All were single bands of the appropriate size. Lane 1: {lower case lambda DNA-HindIII digest standards; Lane 2: empty; Lane 3: 16.2 Kb mtDNA (arrow), control; Lane 4: 16.2 Kb mtDNA (arrow), 2.5 µM rotenone treatment; Lane 5: 7.5 Kb mtDNA (arrow), control; Lane 6: 7.5 Kb mtDNA (arrow), 2.5 µM rotenone treatment. (0.10 MB TIF) [file pone.0004160.s001.tif]

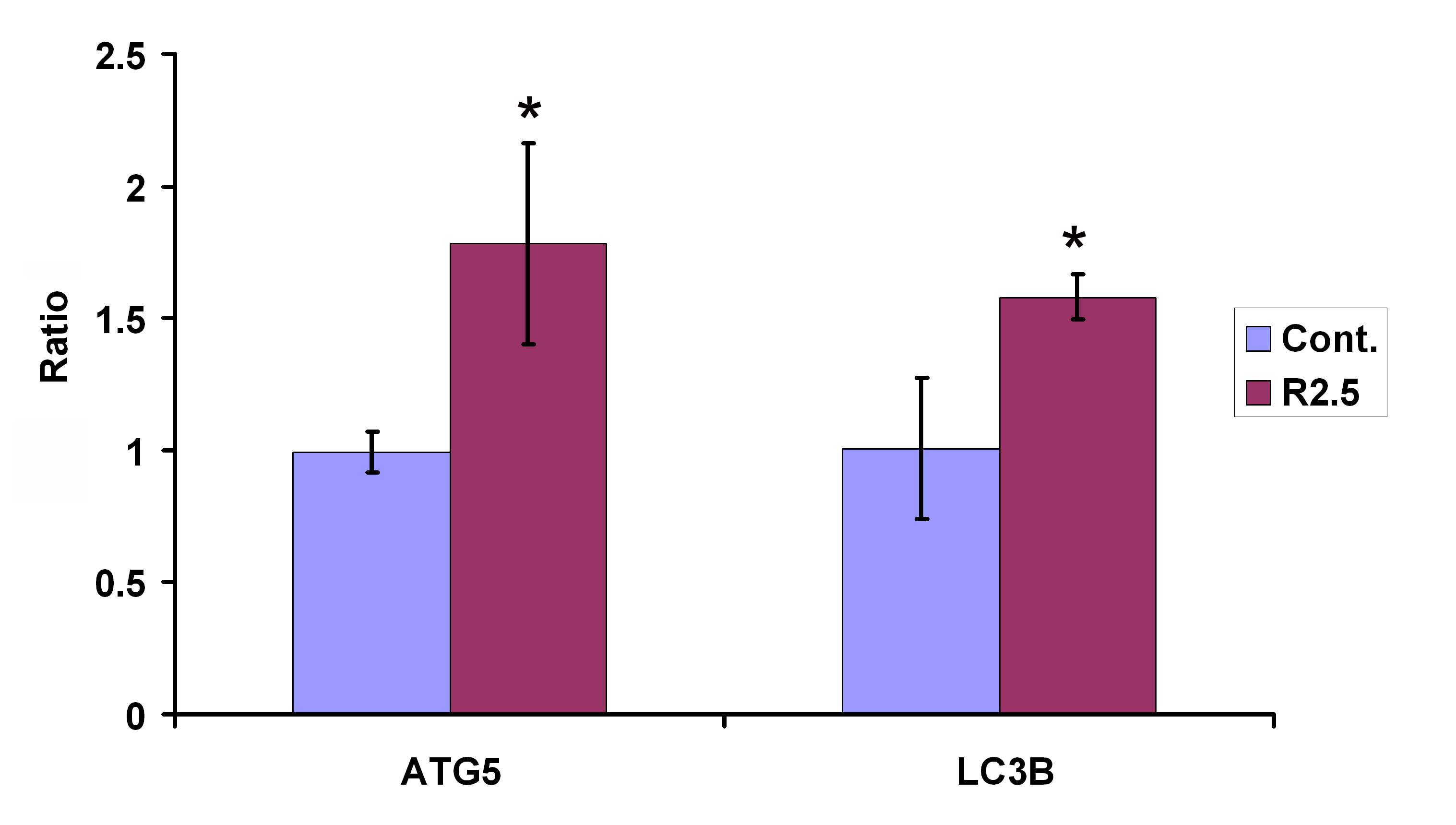

Supplement: Figure S2 — Expression of Atg5 and LC3B in ARPE-19 cells. The differences in expression levels were determined by multiple scans of blots to ensure a maximium and minimum response range for the measured areas and the integrated areas of the bands were calculated by using Image-J software. Data are expressed as normalized ratios to actin. Values are the mean±SEM. Appropriate background subtraction and normalization of the data to actin was done for each blot. There were significant increases in 2.5 µM rotenone treatment of Atg5 (p<0.05, n = 3) and LC3B (p<0.05, n = 3), compared to normal controls. (0.13 MB TIF) [file pone.0004160.s002.tif]

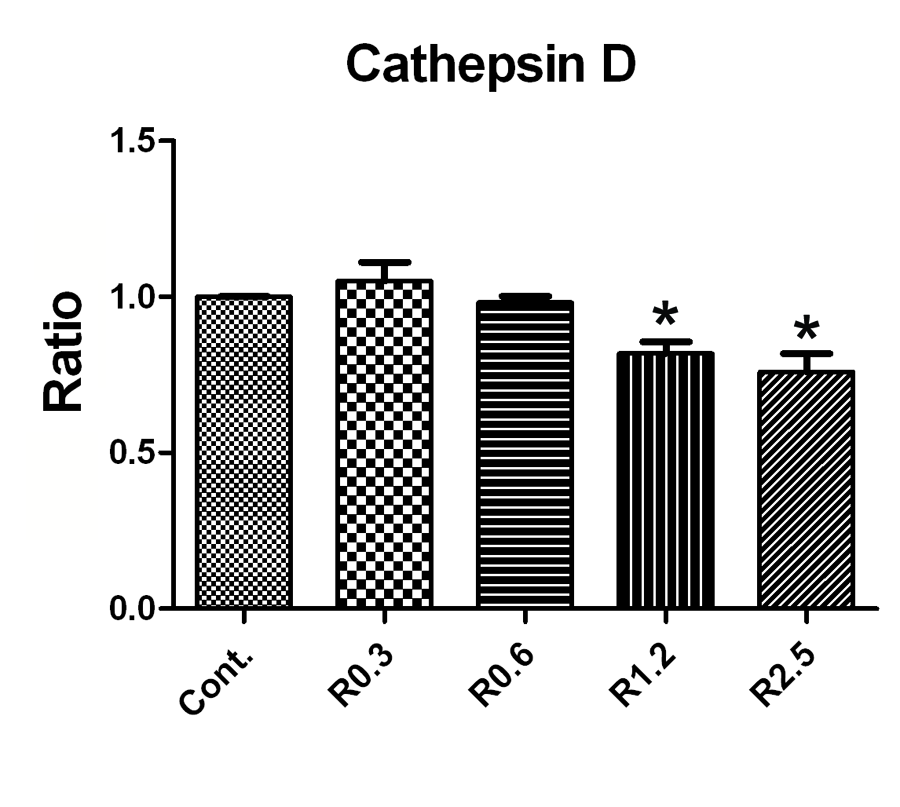

Supplement: Figure S3 — Expression of cathepsin D in ARPE-19 cells. The differences in expression levels of cathepsin D were determined by scanning gels and determining the integrated areas of the bands using Image-J software. Data are expressed as normalized ratios to actin. Values are the mean±SEM. Appropriate background subtraction and normalization of the data to actin was done for each blot. There were significant decreases in 1.25 and 2.5 µM rotenone treatment of cathepsin D (p<0.05, n = 5), compared to normal controls. (2.93 MB TIF) [file pone.0004160.s003.tif]
